# Supplementary material for: The effect of Dexmedetomidine on remote organ injury in the lung in rats with pancreatic ischemia reperfusion model
Source: BMC Anesthesiol. 2025 Oct 17;25:504. doi: 10.1186/s12871-025-03373-8 (PMC12532922; doi:10.1186/s12871-025-03373-8)
Supplement: Supplementary file 1 — Supplementary Material 1. [file 12871_2025_3373_MOESM1_ESM.docx]

**The effect of dexmedetomidine on remote organ injury in the lung in rats with pancreatic ischemia reperfusion model**

**^1^Feyza Aktepe, ^1^Gülay Kip, ^1^Selin Erel, ^2^Ayşegül Küçük, ^1,3,4^Mustafa Arslan, ^5^Şaban Cem Sezen, ^5^Muharrem Atlı, ^6^Hasan Bostancı, ^6^Kürşat Dikmen, ^7^Fatma Er, ^7^Mustafa Kavutçu**

^1^Gazi University Faculty of Medicine Department of Anesthesiology and Reanimation, Ankara, Turkey

^2^Kutahya Health Sciences University Faculty of Medicine, Department of Physiology, Kutahya, Turkey

^3^Gazi University, Life Sciences Application and Research Center, Ankara, Turkey

^4^Gazi University, Laboratory Animal Breeding and Experimental Researches Center (GÜDAM), Ankara, Turkey

^5^Kırıkkale University Faculty of Medicine, Department of Histology and Embryology, Kırıkkale, Turkey

^6^Gazi University Faculty of Medicine, Department of General Surgery, Ankara, Turkey

^7^Gazi University Faculty of Medicine, Department of Medical Biochemistry, Ankara, Turkey

Corresponding Author: Dr. Mustafa Arslan

Gazi University, Medical Faculty,

Department of Anesthesiology and Reanimation

06510, Ankara-Türkiye

GSM: 90 533 422 85 77

E-mail: mustafaarslan@gazi.edu.tr

**ABSTRACT**

**Introduction:** Pancreatic ischemia-reperfusion (IR) injury can trigger acute lung injury by causing remote organ involvement. Dexmedetomidine has been reported to exhibit antioxidant and cytoprotective effects in various organs. This study aimed to evaluate the protective effects of dexmedetomidine on lung tissue at the histopathological and biochemical levels in an experimental pancreatic IR model.

**Methods:** A total of 24 male Wistar-Albino rats were randomly divided into four groups: Sham, Sham + Dexmedetomidine, Ischemia-Reperfusion (IR), and IR + Dexmedetomidine (IR-D). Pancreatic ischemia was induced in the IR and IR-D groups by clamping the inferior splenic and gastroduodenal arteries. In the dexmedetomidine groups, the drug was administered intraperitoneally. Lung tissues were examined using hematoxylin-eosin staining; neutrophil infiltration, alveolar wall thickness, and total injury score were calculated. Levels of thiobarbituric acid reactive substances (TBARS), enzyme activities of catalase, glutathione-S-transferase (GST), and arylesterase were measured as markers of oxidative stress.

**Results:** The IR group exhibited significantly greater neutrophil infiltration/aggregation compared to the Sham group (p = 0.002) and the Sham + Dexmedetomidine group (p = 0.05). Alveolar wall thickness was significantly increased in the IR group compared to both the Sham and Sham + Dexmedetomidine groups (p < 0.001 for both). The total lung injury score was markedly higher in the IR group than in the Sham and Sham + Dexmedetomidine groups (p < 0.001). In the IR-D group, alveolar wall thickness (p = 0.032) and total injury score (p = 0.037) were significantly reduced compared to the IR group. TBARS levels were significantly elevated in the IR group (p = 0.001), while a significant reduction was observed in the IR-D group (p = 0.025). Catalase and arylesterase activities were lower in both IR and IR-D groups compared to the control, but dexmedetomidine significantly increased catalase (p = 0.045) and arylesterase (p = 0.018) activities compared to the IR group.

**Conclusion:** Dexmedetomidine significantly reduced oxidative stress, alleviated alveolar structural damage, and decreased the total injury score in lung tissue in a pancreatic IR model. These findings suggest that dexmedetomidine may be a potential pharmacological agent for preventing pulmonary complications that can arise following pancreatic surgery or severe pancreatitis.

**Keywords:** pancreas, ischemia-reperfusion, dexmedetomidine, lung, rat

**INTRODUCTION**

Ischemia-reperfusion (IR) injury is a potentially serious clinical issue that may arise during various medical and surgical interventions, such as thrombolytic therapy, organ transplantation, coronary angioplasty, and cardiopulmonary bypass. The fundamental pathophysiology of this condition is associated with microvascular dysfunction that develops following the reperfusion of ischemic tissues [1]. During hypoxia and ischemia, anaerobic metabolism is activated in the mitochondria, leading to disruption of the electron transport chain and a subsequent decrease in ATP production. This energy deficit results in the dysfunction of ion channels, intracellular accumulation of sodium, hydrogen, and calcium, and leads to cellular edema and impaired enzyme activity. With reperfusion, mitochondrial damage and electrolyte imbalance contribute to the increased production of reactive oxygen species via systems such as NADPH oxidase, nitric oxide synthase, and xanthine oxidase. Free oxygen radicals trigger cell death through mechanisms such as autophagy, mitoptosis, necrosis, necroptosis, and apoptosis [2-4].

The IR process can affect not only the ischemic organs but also distant organs. This condition is referred to as "remote organ injury" and typically arises as part of the systemic inflammatory response [5-7]. In this process, cytokines, arachidonic acid derivatives, platelet-activating factors, the complement system, increased free oxygen radicals, and neutrophil infiltration play significant roles [8-11]. This systemic response can progress to multiple organ dysfunction syndrome [5]. The pancreas is highly sensitive to oxidative stress generated during this process, which may lead to the development of acute pancreatitis [5]. Conditions such as hemorrhage, septic or hypovolemic shock, cardiac dysfunction, transplantation surgery, vascular embolism, arterial bypass, and surgeries requiring vascular clamping render the pancreas susceptible to IR injury [5].

The lungs are among the organs most affected by this process due to their large reservoirs of monocytes, macrophages, and polymorphonuclear leukocytes, as well as their constant exposure to the external environment [12]. In the context of remote organ injury, pulmonary lesions may result in a clinical spectrum ranging from mild acute lung injury (ALI) to severe respiratory failure and acute respiratory distress syndrome (ARDS) [13].

Dexmedetomidine is a lipophilic α2-adrenoceptor agonist that is widely used in intensive care and anesthesia due to its sedative, anxiolytic, and analgesic properties [14]. Various experimental studies have reported that dexmedetomidine exerts antioxidant and cytoprotective effects against IR injury in organs such as the ovaries, testes, kidneys, lungs, and heart [5, 15–21].

This study aimed to investigate the remote organ-protective effects of dexmedetomidine on lung tissue at the histopathological and biochemical levels in a pancreatic IR injury model.

**MATERIALS AND METHODS**

This study was conducted at the Gazi University Experimental Research Center (GÜDAM) after obtaining approval from the Gazi University Animal Experiments Ethics Committee (Approval date: 29.05.2024; Approval code: G.U.ET-24.051). The rats used in the study were obtained from the Gazi University Experimental Research Center (GÜDAM). The study was conducted at the same center in accordance with the ARRIVE 2.0 guidelines. All procedures adhered to the accepted standards outlined in the Guide for the Care and Use of Laboratory Animals.

A total of 24 adult male Wistar-Albino rats, aged 4 months and weighing between 250–300 grams, were used in the study. The rats were housed under controlled conditions with a temperature of 20–21°C and a 12-hour light/12-hour dark cycle. Food and water were provided ad libitum until the beginning of the experiment. The animals were randomly allocated into four groups using a computer-generated random number sequence.

All surgical procedures were performed under sterile conditions and anesthesia induced by ketamine and xylazine. At the beginning of the experiment, anesthesia was achieved via intramuscular injection of 50 mg/kg ketamine hydrochloride (Ketalar® Pfizer PFE İlaçları, İstanbul, Turkey) and 5 mg/kg xylazine hydrochloride (Alfazyne, 2%, Ege Vet). Procedures were performed with the rats in the supine position under a warming lamp. Following a midline laparotomy, the pancreas and spleen were accessed inferior to the stomach. In the IR model, the short gastric arteries were dissected and ligated using in surgical lopues (3.5x, Design for vision, Inc, Newyork, USA) and the greater omentum was detached from the greater curvature of the stomach. Ischemia was induced by clamping the inferior splenic artery and the gastroduodenal artery. At the end of the ischemia period, the vascular clamps were removed. At the conclusion of the reperfusion period in all rats, a midline laparotomy was repeated; they were anesthetized with ketamine (50 mg/kg) and xylazine (10 mg/kg) and then euthanized by collecting blood (5–10 ml) via intracardiac blood sampling under anesthesia. After their heartbeats and respiration ceased, the rats were monitored for a further 2 min to confirm death. Following euthanasia, lung tissues were carefully removed without shaking or disturbing their integrity. The right lung tissues were fixed in 10% formalin for histopathological examination. The left lung tissues were preserved at −80°C for oxidant status analysis.

**Sham Group (n = 6):**

In this group, a midline abdominal laparotomy was performed under anesthesia without induction of ischemia. The inferior splenic artery was exposed but neither ligation nor occlusion was carried out. At 180 minutes post-procedure, the rats were sacrificed under anesthesia via abdominal blood collection, and lung tissues were harvested. Histological and biochemical evaluations were conducted on these tissues.

**Sham-Dexmedetomidine Group (Group D, n = 6):**

In this group, without inducing ischemia, dexmedetomidine was administered intraperitoneally at a dose of 100 µg/kg. Thirty minutes later, under anesthesia, only a midline abdominal laparotomy was performed, and the inferior splenic artery was exposed neither ligation nor occlusion was carried out. At 180 minutes post-procedure, the rats were sacrificed under ketamine anesthesia via abdominal blood collection, and lung tissues were harvested. Histological and biochemical evaluations were conducted on these tissues.

**Ischemia-Reperfusion Group (Group IR, n = 6):**

In this group, under ketamine and xylazine anesthesia, a midline abdominal laparotomy was performed. The short gastric arteries were dissected and ligated, and the greater omentum was detached from the greater curvature of the stomach. To induce complete pancreatic ischemia, the inferior splenic artery and the gastroduodenal artery were clamped. Following 60 minutes of ischemia and 120 minutes of reperfusion, the rats were sacrificed under anesthesia via abdominal blood collection, and lung tissues were harvested for histological and biochemical evaluations.

**Ischemia-Reperfusion-Dexmedetomidine Group (Group IR-D, n = 6):**

In this group, dexmedetomidine was administered intraperitoneally at a dose of 100 µg/kg, 30 minutes prior to the induction of ischemia. Following ketamine and xylazine anesthesia, a midline abdominal laparotomy was performed. The short gastric arteries were dissected and ligated, and the greater omentum was detached from the greater curvature of the stomach. To induce complete pancreatic ischemia, the inferior splenic artery and the gastroduodenal artery were clamped. After 60 minutes of ischemia and 120 minutes of reperfusion, the rats were sacrificed under anesthesia via abdominal blood collection, and lung tissues were harvested for histological and biochemical evaluations. The selected dose and timing of dexmedetomidine administration, as well as the ischemia–reperfusion protocol, were based on previously published experimental studies (22,23).

**Histopathological Evaluation**

Histopathological examination was performed at Kırıkkale University, Faculty of Medicine, Department of Histology and Embryology. Lung tissues obtained at the end of the experiment were fixed in 10% neutral formalin for 72 hours, followed by routine tissue processing and embedding in paraffin blocks. Sections of 4–5 µm thickness were prepared from the blocks and stained with hematoxylin and eosin. Histopathological evaluation was performed in 10 randomly selected fields per section under a light microscope by an investigator blinded to group allocation. Two main parameters were examined in these fields: neutrophil infiltration/aggregation and alveolar wall thickness. Each parameter was scored separately using a 4-point scale according to the severity of damage (0 = none, 1 = mild, 2 = moderate, 3 = severe). The sum of the scores from these two parameters was considered the total lung injury score for each field [24].

**Biochemical Evaluation**

To assess oxidative stress activity in the lung tissue, the following biochemical parameters were analyzed: glutathione S-transferase (GST), catalase, and arylesterase enzyme activities, TBARS levels. Biochemical analyses were performed by investigators blinded to group allocation.

**Statistical Analysis**

Statistical analysis was performed using the SPSS version 26.0 software package, and a p-value < 0.05 was considered statistically significant. The conformity of the data to normal distribution was evaluated with the Shapiro–Wilk test. All data are expressed as the mean ± standard error of the mean (SEM) or median (IQR). Comparisons of >2 groups were performed using the Kruskal–Wallis test followed by Dunn’s post hoc test or one-way ANOVA followed by Tukey’s post hoc test.

**RESULTS**

Neutrophil infiltration/aggregation was found to differ significantly among the groups (p = 0.008). The IR group exhibited significantly greater neutrophil infiltration/aggregation compared to the Sham and D groups (p = 0.002 and p = 0.05, respectively) (Table 1, Figures 1–8).

Alveolar wall thickness also showed a significant difference among the groups (p = 0.001). The IR group demonstrated increased alveolar wall thickness compared to the Sham and D groups (p < 0.001 for both). Moreover, the IR-D group exhibited significantly lower alveolar wall thickness compared to the IR group (p = 0.032) (Table 1, Figures 1–8).

The total injury score differed significantly among the groups (p = 0.001). The IR group showed a significantly higher total injury score compared to the Sham and D groups (p < 0.001 for both). The IR-D group had a significantly lower total injury score compared to the IR group (p = 0.037) (Table 1, Figures 1–8).

A statistically significant difference in TBARS levels was found among the groups (p = 0.004). Post hoc analyses showed that TBARS levels in the IR group were significantly higher than in both the Sham and D groups (p = 0.001 for both). In contrast, TBARS levels were significantly lower in the IR-D group compared to the IR group (p = 0.025) (Table 2).

When comparing catalase enzyme activity among the groups, a significant difference was observed (p < 0.001). Catalase activity was significantly lower in the IR and IR-D groups compared to the Sham group (p < 0.0001). Similarly, catalase activity was significantly lower in the IR and IR-D groups compared to the D group (p < 0.001 and p = 0.010, respectively). However, catalase activity in the IR-D group was significantly higher than in the IR group (p = 0.045) (Table 2).

GST enzyme activity also differed significantly among the groups (p = 0.022). GST activity was significantly lower in the IR and IR-D groups compared to the Sham group (p = 0.003 and p = 0.025, respectively) (Table 2).

A significant difference in arylesterase enzyme activity was found among the groups (p < 0.001). Arylesterase activity was significantly lower in both the IR and IR-D groups compared to the Sham group (p < 0.001 for all). Additionally, arylesterase activity was significantly lower in the IR and IR-D groups compared to the D group (p < 0.001 for both). However, arylesterase activity was significantly higher in the IR-D group compared to the IR group (p = 0.018) (Table 2).

**DISCUSSION**

In our study, dexmedetomidine administration was found to reduce alveolar wall thickness, neutrophil infiltration, and the total lung injury score. Additionally, dexmedetomidine significantly decreased TBARS levels, a marker of lipid peroxidation, and partially preserved GST activity, indicating its effectiveness in mitigating oxidative stress. Furthermore, the activities of catalase and arylesterase—key components of the antioxidant defense system—were significantly increased with dexmedetomidine treatment. These findings suggest that lung injury developing secondary to pancreatic IR injury may be alleviated through the antioxidant and anti-inflammatory effects of dexmedetomidine.

Pancreatitis is an acute inflammatory disease associated with high morbidity and mortality rates and may be complicated by the involvement of distant organs [25]. The lungs are among the organs most commonly affected in the early phase [24]. To mitigate pancreatitis induced by ischemia-reperfusion, as well as remote organ injuries such as ALI and ARDS that may result from pancreatitis, various pharmacological and physical strategies have been investigated. These include ischemic preconditioning, antioxidants, free radical scavengers, and anesthetic agents [25, 27–29].

In IR studies, TBARS, catalase, GST, and arylesterase are commonly used biochemical parameters. During IR injury, reactive oxygen metabolites, due to their high reactivity, attack membrane lipids, leading to lipid peroxidation, and also oxidize cellular proteins and DNA, thereby triggering tissue damage [30]. Among the widely used methods for detecting oxidative stress is the TBARS assay [31]. Therefore, in our study, we measured TBARS concentrations in lung tissue to determine the level of lipid peroxidation. The literature indicates that TBARS levels significantly increase following IR injury. For instance, in a study investigating the protective effect of chrysin in a rat model of myocardial IR, TBARS levels were markedly elevated in the IR group compared to controls, while chrysin pretreatment significantly reduced this elevation [32]. Similarly, Jun Li et al. evaluated the antioxidant effects of anthocyanins in renal IR and reported significantly higher TBARS levels in the IR group compared to controls, which were notably reduced with anthocyanin administration [33]. Our findings are consistent with these results. TBARS levels differed significantly among groups; in the IR group, TBARS levels were markedly higher than in both the Sham and dexmedetomidine groups. In contrast, the group that received prophylactic dexmedetomidine treatment prior to IR showed significantly lower TBARS levels compared to the IR group. These results suggest that dexmedetomidine may protect lung tissue from oxidative damage by limiting IR-induced lipid peroxidation.

Catalase is one of the primary enzymes that, in cooperation with superoxide dismutase, converts hydrogen peroxide (H₂O₂) into water and oxygen; elevated circulating levels reflect the efficiency of the organism’s antioxidant defense system [34]. Studies have shown that catalase activity is suppressed during IR injury. In a cerebral IR model, Chao et al. observed reduced catalase levels in the IR group compared to the control group, and reported that troxerutin treatment significantly increased catalase activity [35]. Similarly, in a testicular IR model, ellagic acid therapy was found to restore catalase activity, while catalase activities were markedly reduced in the IR group compared to both the control and IR + ellagic acid groups [36]. In our study, catalase enzyme activities were significantly lower in the IR group compared to the sham group, whereas dexmedetomidine administration was found to increase catalase enzyme activities.

GST is a multifunctional enzyme with broad activity and specific substrates, found in many human tissues. Animal studies have shown that GST activity decreases during IR injury [37]. Several studies in the literature confirm this reduction in antioxidant capacity following IR. For instance, in rat models of renal and hepatic IR, GST enzyme activities were significantly reduced in I/R groups compared to controls, while pharmacological treatments such as avanafil and rimonabant were shown to reverse this decline [38, 39]. Similarly, Wang et al. reported that dexmedetomidine treatment significantly increased GST enzyme activities in lung tissue in a lower extremity IR model [40]. The findings of our study are partially consistent with the literature; GST enzyme activity was significantly lower in both the IR and IR-D groups compared to the Sham group. However, unlike previous studies, GST activity in the IR-D group did not show a statistically significant increase compared to the IR group. This discrepancy is primarily attributed to methodological differences between experimental models.

Serum paraoxonase (PON1) is a calcium-dependent esterase hydrolase associated with high-density lipoprotein (HDL) and exhibits two primary enzymatic activities: paraoxonase and arylesterase. Arylesterase activity is considered important as it reflects the functional mass of the enzyme [41]. The primary physiological role of PON1 is to protect against lipid peroxidation by preventing the oxidation of both low-density lipoprotein (LDL) and HDL. A strong negative correlation has been reported between PON1 activity and markers of oxidative stress [42, 43]. Consistent with this protective function, experimental IR models have demonstrated a marked reduction in both PON1 and arylesterase activities [44]. In our study, the increase in arylesterase activity observed with dexmedetomidine treatment suggests that the drug not only exerts a general antioxidant effect but also preserves or restores this specific and critical HDL-associated enzymatic defense mechanism. This finding indicates that dexmedetomidine may play a role in mitigating IR-induced oxidative stress.

The literature demonstrates that dexmedetomidine exerts multifaceted protective effects against IR injury in various organs. These effects are primarily attributed to its ability to enhance antioxidant capacity while reducing oxidative damage and apoptosis [16]. For example, in myocardial IR injury, dexmedetomidine has been shown to inhibit oxidative stress and cell death by modulating signaling pathways such as Trx1/Akt and p75NTR/p38MAPK/JNK [17, 18]. Similarly, in a hepatic IR model, it exerted protective effects by activating the PI3K/AKT/Nrf2 pathway and suppressing the NLRP3 inflammasome, whereas in cardiac tissue, it was found to regulate bradykinin receptor expression to confer protection [19, 20].

Despite the well-documented and potent protective potential of dexmedetomidine, studies investigating its effects on pancreatic IR injury remain limited. The existing literature on pancreatic IR injury primarily focuses on the cytoprotective effects of antioxidant and anti-apoptotic agents such as melatonin, or molecules that regulate microvascular perfusion, such as bradykinin antagonists [5, 21]. In this context, our study was designed to address this gap by evaluating whether the strong antioxidant and organ-protective properties of dexmedetomidine, which have been demonstrated in various tissues, are also effective against pancreatic IR injury. In terms of clinical applicability, the dose and timing of dexmedetomidine used in this experimental model do not fully correspond to clinical practice, particularly considering its sedative and hemodynamic effects in humans. In addition, although catalase and arylesterase activities improved with treatment, GST activity did not show a significant recovery. This may be related to differences in enzyme regulation or to limitations of the experimental model.

**Limitations**

Although our study employed both biochemical and histopathological analysis techniques, certain limitations were present. The relatively small sample size without a priori power analysis, the use of a single time-point assessment, and the lack of inter-observer reproducibility may limit the generalizability of the findings. In addition, the lung injury score was restricted to neutrophil infiltration/aggregation and alveolar wall thickness, without assessment of other features such as edema, hemorrhage, hyaline membranes, or alveolar exudate. The biomarker scope was limited, and no statistical correction for multiple comparisons (e.g., Holm–Bonferroni or FDR) was applied.

**Conclusion**

In conclusion, our study demonstrated that dexmedetomidine has a protective effect through its antioxidant properties in a rat model of experimental pancreatic IR injury. This was evidenced by improvements in lung histopathological findings (lung injury score, neutrophil infiltration, alveolar wall thickness), as well as in biochemical parameters including arylesterase, catalase, and GST enzyme activities, and TBARS levels. These findings indicate that dexmedetomidine may have a protective role against pancreatic IR-induced pulmonary injury; however, the translational relevance remains limited by the experimental nature of the study.

**Funding**

No funding was received.

**Availability of data and materials**

The datasets used and/or analyzed during the current study are available from the corresponding author on reasonable request.

**Authors’ contributions**

MA, FA, KD, AK, GK, and HB designed the study, and analyzed and interpreted data. FA, HB and KD performed the experiments. KD, SE, FA, and MK confirm the authenticity of all the raw data. GK, MK, MA and ŞCS provided scientific and technical assistance, and critically revised the article for important intellectual content. FA and FE collected samples. MK, FE, MA and ŞCS performed biochemical and histopathological experiments. All authors have read and approved the final manuscript.

**Ethics approval and consent to participate**

Ethical approval for the study was obtained from Animal Research Committee of Gazi University (Approval date: 29.05.2024; Approval code: G.U.ET-24.051). Clinical trial number: not applicable.

Consent to participate: Not applicable.

**Patient consent for publication**

Not applicable.

**Competing interests**

The authors declare that they have no competing interests.

**Table 1.** Lung tissue histopathological findings [Median (IQR)]

|  | **Group Sham**  **(n=6)** | **Group D**  **(n=6)** | **Group IR**  **(n=6)** | **Group IR-D**  **(n=6)** | **P**** |
| --- | --- | --- | --- | --- | --- |
| **Neutrophil infiltration/aggregation** | 0,00 (0-1) | 0,50 (0-1) | 1,50 (1-2,5)*,& | 1,00 (0,75-1,25) | 0.008 |
| **Alveolar wall thickness** | 0,50 (0-1) | 0,50 (0-1) | 2,00 (1-3)*,& | 1,00 (1-1,25)+ | 0.001 |
| **Total damage score** | 0,50 (0-2) | 1,00 (0-2) | 4,00 (2-4)*,& | 2,00 (1,75-2,5)+ | 0.001 |

p**: Significance level with Kruskal Wallis test p< 0.05

*p<0.05: Compared to Group Sham

&p<0.05: Compared with Group D

+p<0.05: Compared with Group IR

**Table 2.** Lung tissue oxidant status parameters [Mean ± SEM]

|  | **Group Sham**  **(n=6)** | **Group D**  **(n=6)** | **Group IR**  **(n=6)** | **Group IR-D**  **(n=6)** | **P**** |
| --- | --- | --- | --- | --- | --- |
| **TBARS (nmol/mg.pro)** | 9.34±0.31 | 9.53±0.60 | 16.87±1.73*.& | 12.05±2.12+ | 0.004 |
| **Catalase (IU/mg.pro)** | 75.62±9.68 | 63.38±5.30 | 20.73±2.79*.& | 38.78±4.69*.&.+ | <0.001 |
| **GST (IU/mg.pro)** | 2.34±0.33 | 1.66±0.31 | 1.17±0.08* | 1.51±0.19* | 0.022 |
| **Arylesterase (IU/mg.pro)** | 2.72±0.29 | 2.29±0.15 | 0.55±0.07*.& | 1.22±0.16*.&.+ | <0.001 |

p**: Significance level with ANOVA test p< 0.05

*p<0.05: Compared to Group Sham

&p<0.05: Compared with Group D

+p<0.05: Compared with Group IR

**Figures**


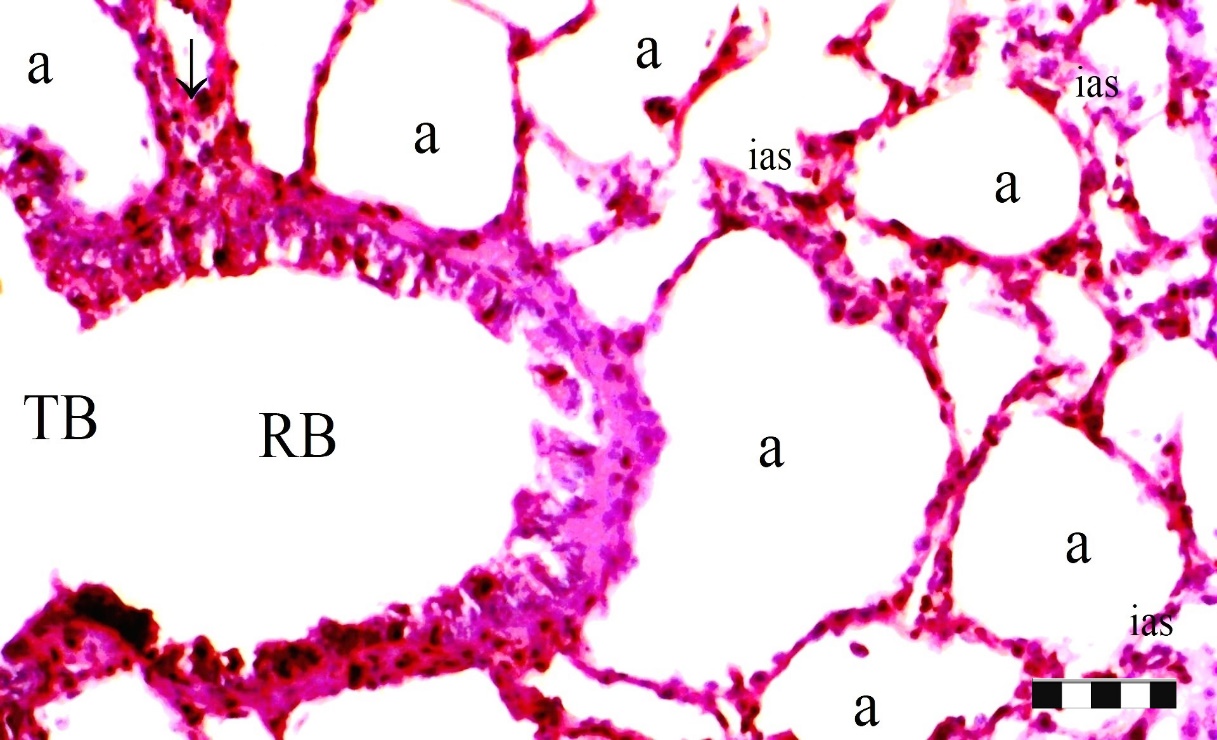


**Figure 1.** Group Sham; a: alveoli, RB: respiratory bronchiole, TB: terminal bronchiole, ias: inter alveolar septum (X100), scale bar 100 µm


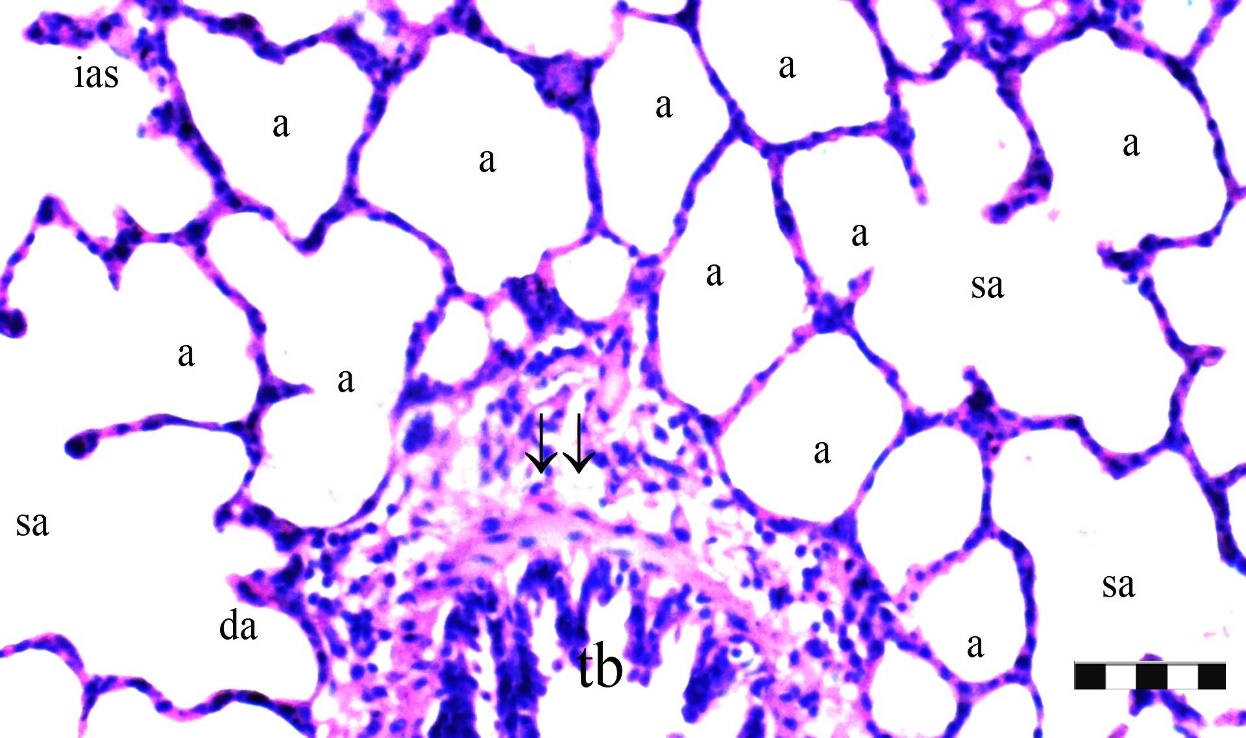


**Figure 2.** Group Sham; a: alveoli, tb: terminal bronchiole, da: ductus alveolaris, sa: saccus alveolaris, ias: inter alveolar septum, ↓↓ : thickening (X100), scale bar 100 µm


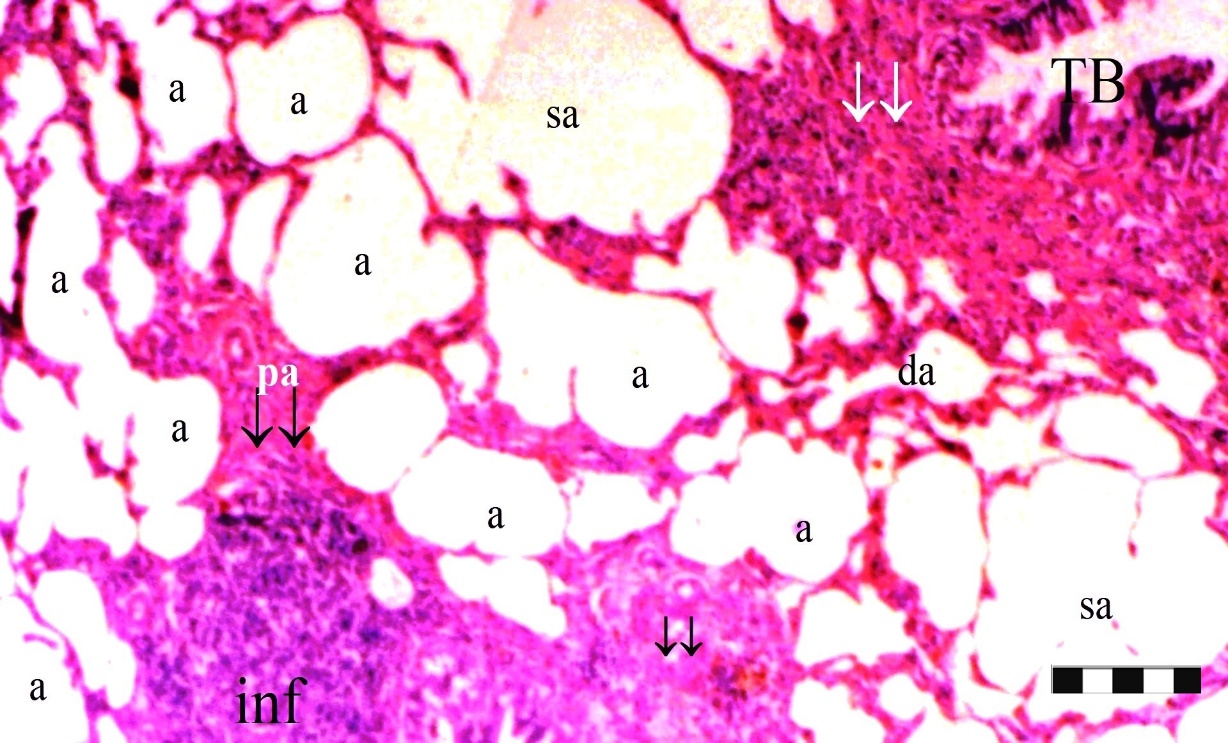


**Figure 3.** Group D; a: alveoli, TB: terminal bronchiole, pa: pulmonary artery branches, da: ductus alveolaris, sa: saccus alveolaris, ↓↓ : thickening, inf: inflammation (X100), scale bar 100 µm

*
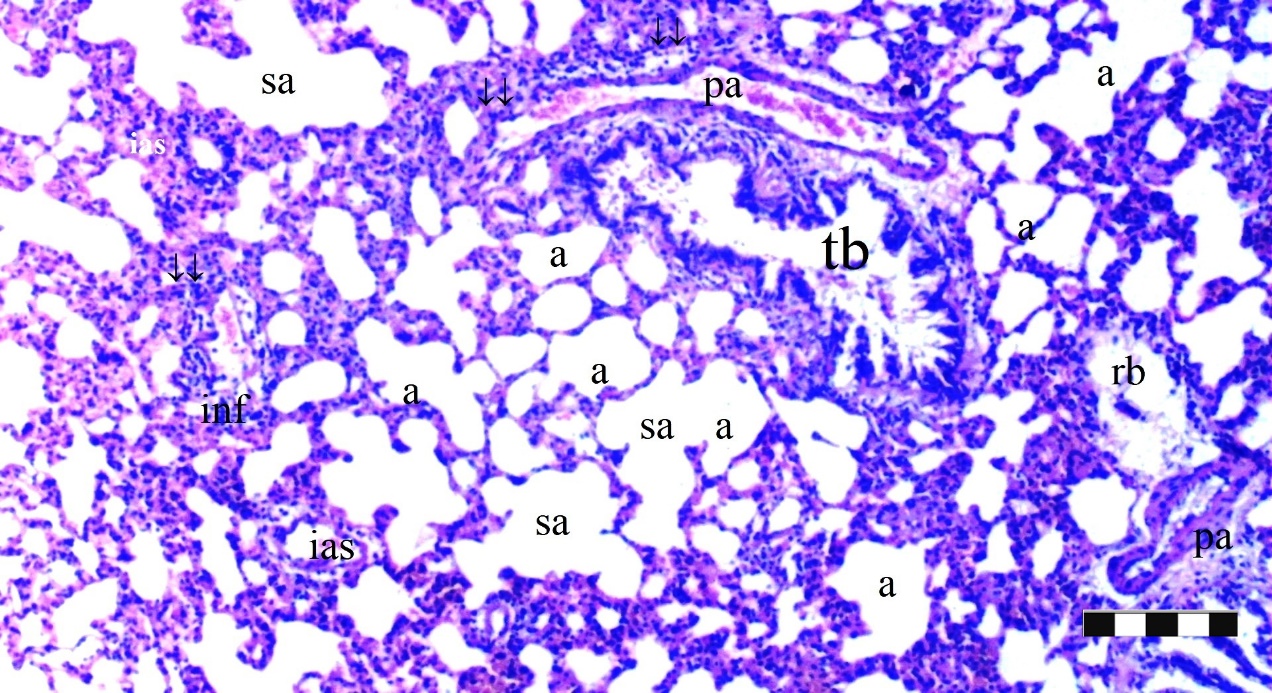
*

**Figure 4.** Group D; a: alveoli, rb: respiratory bronchiole, tb: terminal bronchiole, pa: pulmonary artery branches, sa: saccus alveolaris, ias: inter alveolar septum, ↓↓ : thickening, inf: inflammation (X40), scale bar 100 µm


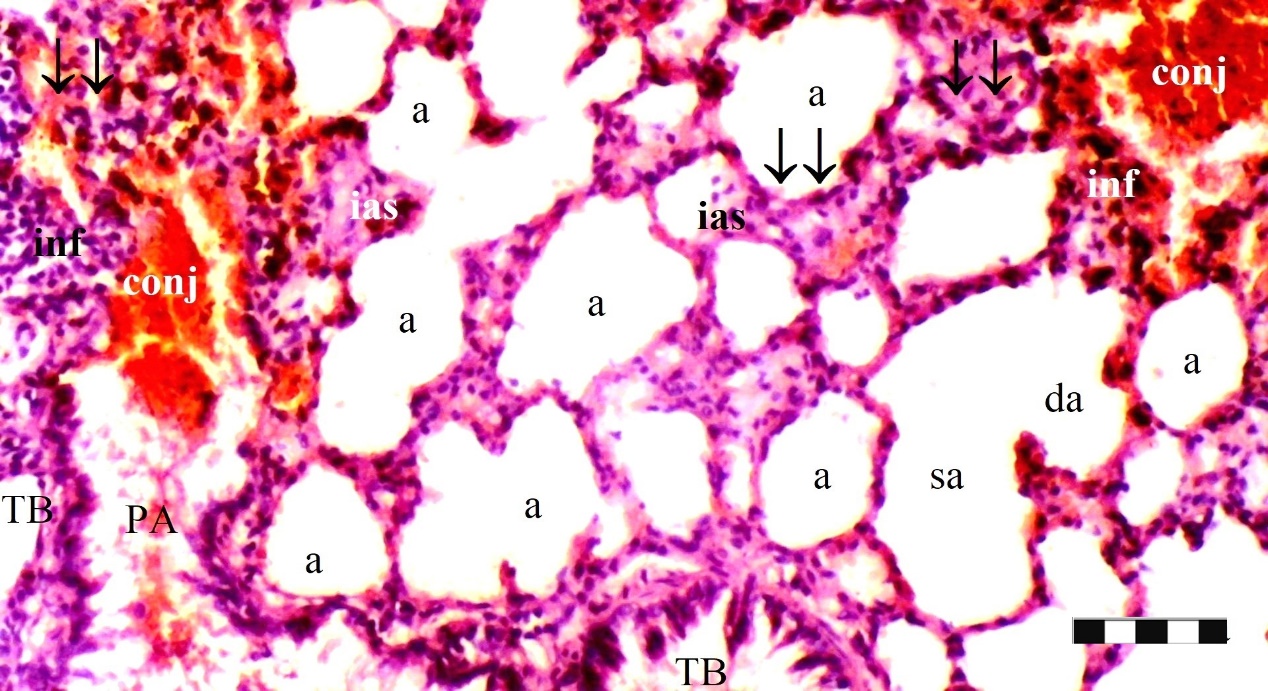


**Figure 5.** Group IR; a: alveoli, TB: terminal bronchiole, PA: pulmonary artery branches, da: ductus alveolaris, sa: saccus alveolaris, ias: inter alveolar septum, ↓↓ : thickening, inf: inflammation, conj: congestion (X100), scale bar 100 µm


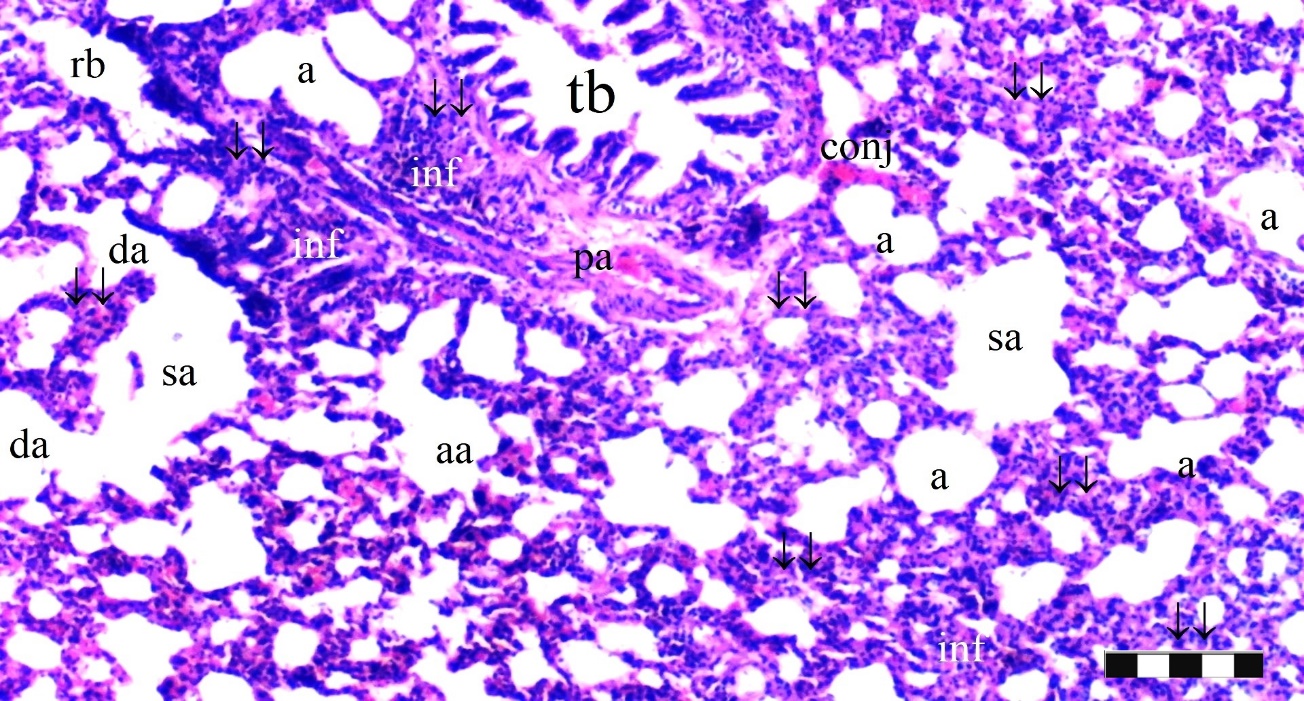


**Figure 6.** Group IR; a: alveoli, rb: respiratory bronchiole, tb: terminal bronchiole, pa: pulmonary artery branches, da: ductus alveolaris, sa: saccus alveolaris, ↓↓ : thickening, inf: inflammation, conj: congestion (X40), scale bar 100 µm


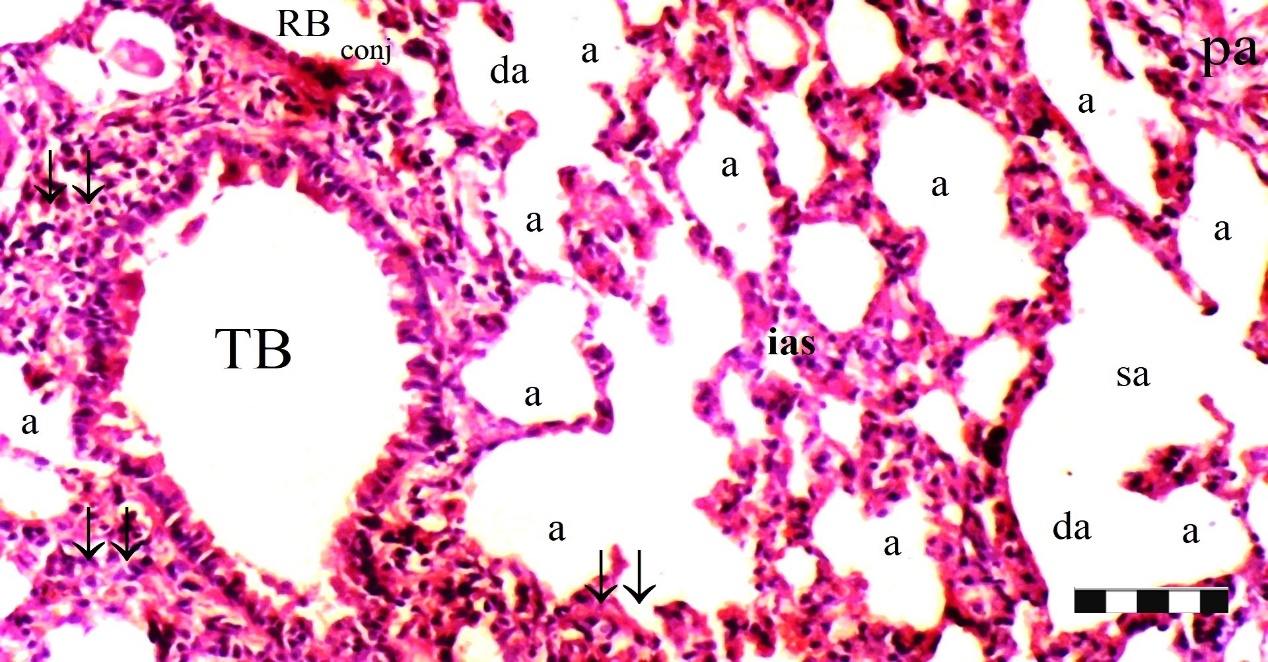


**Figure 7.** Group IR-D; a: alveoli, RB: respiratory bronchiole, TB: terminal bronchiole, pa: pulmonary artery branches, da: ductus alveolaris, sa: saccus alveolaris, ias: inter alveolar septum, ↓↓ : thickening, conj: congestion (X100), scale bar 100 µm

**
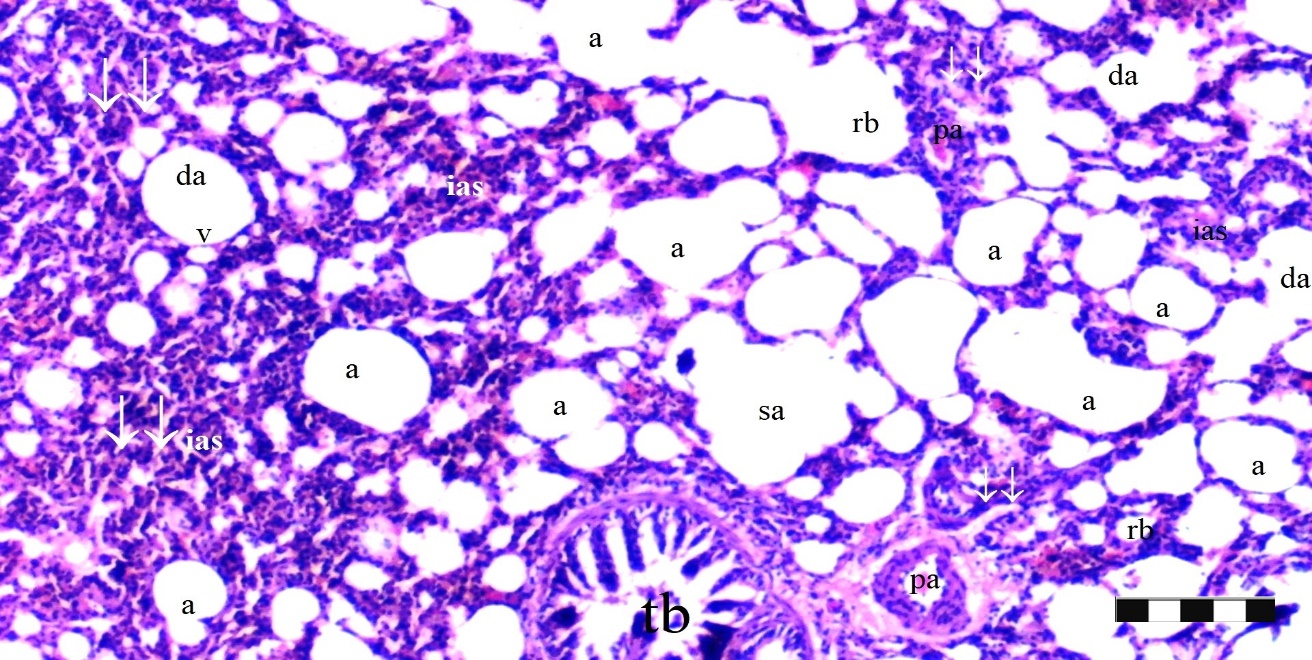
**

**Figure 8.** Group IR-D; a: alveoli, rb: respiratory bronchiole, tb: terminal bronchiole, pa: pulmonary artery branches, da: ductus alveolaris, sa: saccus alveolaris, ias: inter alveolar septum, ↓↓ : thickening, v: emphysematous alveolar vacuole (X40), scale bar 100 µm

**References**

1. Teke, Z., B. Kabay, and Ö. Akın, İskemi-reperfüzyon hasarının patofizyolojisi. Pamukkale Tıp Dergisi, 2008.

2. Wu, M.-Y., et al., Current mechanistic concepts in ischemia and reperfusion injury. Cellular Physiology and Biochemistry, 2018. 46(4): p. 1650-1667.

3. Ozturk L, et al. Effect of different doses of pregabalin on skeletal muscle ischaemia-reperfusion injury in rats. Bratisl Med J 118 (7) 417–422,(2017).  DOI: [10.4149/BLL_2017_081](http://doi.org/10.4149/BLL_2017_081)

4. Özer A, et al. Protective effect of erdosteine on erythrocyte deformability in a rat model of lower limb ischemia/reperfusion injury. Turk J Med Sci 48: 187-190, (2018). DOI: [10.3906/sag-1706-160](http://doi.org/10.3906/sag-1706-160)

5. Muñoz‐Casares, F.C., et al., Melatonin reduces apoptosis and necrosis induced by ischemia/reperfusion injury of the pancreas. Journal of Pineal Research, 2006. 40(3): p. 195-203.

6. Demirtas, H., et al. Protective Effects of BPC157onLiver,Kidney,andLung Distant OrganDamageinRatswith Experimental Lower-Extremity Ischemia–Reperfusion Injury. Medicina 2025, 61, 291. https:// doi.org/10.3390/medicina61020291

7. Özer A , et al. Evaluation of the effect of enriched hydrogen saline solution on distant organ (lung) damage in skeletal muscle ischemia reperfusion in rats. Science Progress 2024, Vol. 107(2) 1–13 DOI:10.1177/00368504241257060

8. Isbir, S., et al., Effect of acute lower limb ischemia/reperfusion injury on the free oxygen radical systems in lungs. Turkish J Thorac Cardiovasc Surg, 2000. 8: p. 632-4.

9. Rocker, G.M., Ischaemia/reperfusion, inflammatory responses and acute lung injury. Thorax, 1997. 52(10): p. 841.

**10. Demirtaş H,** et al. Protective effects of bosentan via endothelin receptor antagonism in experimental ischemia-reperfusion injury in the lower limb of rats. Drug Des Devel Ther. 2025;19:1561-1573. doi:10.2147/DDDT.S510885

11. Gülcan M.B, et al. Ozone Administration Reduces Myocardial Ischemia Reperfusion Injury in Streptozotocin Induced Diabetes Mellitus Rat Model. Drug Design, Development and Therapy 2024:18 4203–4213. doi.org/10.2147/DDDT.S482309

12. Sivrikoz, M.C., et al., Antioxidant effects of pentoxiflline on the ischemia reperfusion and reexpansion injury of the lung. European Journal of Therapeutics, 2001. 12(1): p. 1-4.

13. Carden, D.L. and D.N. Granger, Pathophysiology of ischaemia-reperfusion injury. J Pathol, 2000. 190(3): p. 255-66.

14. Bhana, N., K.L. Goa, and K.J. McClellan, Dexmedetomidine. Drugs, 2000. 59(2): p. 263-8; discussion 269-70.

15. Hemsinli, D., et al., Dexmedetomidine attenuates pneumocyte apoptosis and inflammation induced by aortic ischemia-reperfusion injury. Clin Exp Hypertens, 2022. 44(7): p. 595-600.

16. Zhang, Z.Z., et al., Effect of dexmedetomidine on ncRNA and mRNA profiles of cerebral ischemia-reperfusion injury in transient middle cerebral artery occlusion rats model. Frontiers in Pharmacology, 2024. 15: p. 1437445.

17. Wu, Z.-l., J.R.J. Davis, and Y. Zhu, Dexmedetomidine Protects against Myocardial Ischemia/Reperfusion Injury by Ameliorating Oxidative Stress and Cell Apoptosis through the Trx1‐Dependent Akt Pathway. BioMed Research International, 2020. 2020(1): p. 8979270.

18. Wang, Z., et al., Dexmedetomidine alleviates lipopolysaccharide‐induced acute kidney injury by inhibiting p75NTR‐mediated oxidative stress and apoptosis. Oxidative medicine and cellular longevity, 2020. 2020(1): p. 5454210.

19. Song, J., et al., Dexmedetomidine protects the heart against ischemia reperfusion injury via regulation of the bradykinin receptors. European Journal of Pharmacology, 2021. 911: p. 174493.

20. Wu, Y., et al., Dexmedetomidine alleviates hepatic ischaemia‐reperfusion injury via the PI3K/AKT/Nrf2‐NLRP3 pathway. Journal of cellular and molecular medicine, 2021. 25(21): p. 9983-9994.

21. Hoffmann, T., et al., Bradykinin antagonists HOE-440 and CP-0597 diminish microcirculatory injury after ischaemia-reperfusion of the pancreas in rats. Journal of British Surgery, 1996. 83(2): p. 189-195.

22. Bostancı, H., Erel, S., Küçük, A., Kip, G., Sezen, Ş. C., Gokgoz, S et al. (2024). Dexmedetomidine’s Effects on the Livers and Kidneys of Rats with Pancreatic Ischemia-Reperfusion Injury. *Drug Design, Development and Therapy*, *18*, 1785–1797.

23. Şengel N, Köksal Z, Dursun AD, Kurtipek Ö, Sezen ŞC, Arslan M, Kavutçu M. Effects of dexmedetomidine administered through different routes on kidney tissue in rats with spinal cord ischaemia–reperfusion injury. Drug Design, Development and Therapy. 2023 Dec 31:2229-39.

24. Tuncay, A., Sivgin, V., Ozdemirkan, A., Sezen, S. C., Boyunaga, H., Kucuk, A., … Arslan, M. (2020). The Effect of Cerium Oxide on Lung Tissue in Lower Extremity Ischemia Reperfusion Injury in Sevoflurane Administered Rats. International Journal of Nanomedicine, 15, 7481–7489. https://doi.org/10.2147/IJN.S263001

25. Ateyya, H., H.M. Wagih, and N.A. El-Sherbeeny, Effect of tiron on remote organ injury in rats with severe acute pancreatitis induced by L-arginine. Naunyn-Schmiedeberg's archives of pharmacology, 2016. 389: p. 873-885.

26. Landahl, P., D. Ansari, and R. Andersson, Severe acute pancreatitis: gut barrier failure, systemic inflammatory response, acute lung injury, and the role of the mesenteric lymph. Surgical infections, 2015. 16(6): p. 651-656.

27. Czakó, L., et al., Oxidative stress in distant organs and the effects of allopurinol during experimental acute pancreatitis. International Journal of Pancreatology, 2000. 27: p. 209-216.

28. Dembiński, A., et al., Ischemic preconditioning reduces the severity of ischemia/reperfusion-induced pancreatitis. European journal of pharmacology, 2003. 473(2-3): p. 207-216.

29. Elder, A.S., G.T. Saccone, and D.-L. Dixon, Lung injury in acute pancreatitis: mechanisms underlying augmented secondary injury. Pancreatology, 2012. 12(1): p. 49-56.

30. Li, C. and R.M. Jackson, Reactive species mechanisms of cellular hypoxia-reoxygenation injury. American Journal of Physiology-Cell Physiology, 2002. 282(2): p. C227-C241.

31. Oakes, K.D. and G.J. Van Der Kraak, Utility of the TBARS assay in detecting oxidative stress in white sucker (Catostomus commersoni) populations exposed to pulp mill effluent. Aquatic Toxicology, 2003. 63(4): p. 447-463.

32. Rani, N. and D.S. Arya, Chrysin rescues rat myocardium from ischemia-reperfusion injury via PPAR-γ/Nrf2 activation. European journal of pharmacology, 2020. 883: p. 173389.

33. Li, L., et al., The protective effect of anthocyanins extracted from Aronia melanocarpa berry in renal ischemia‐reperfusion injury in mice. Mediators of Inflammation, 2021. 2021(1): p. 7372893.

34. Valko, M., et al., Free radicals, metals and antioxidants in oxidative stress-induced cancer. Chemico-biological interactions, 2006. 160(1): p. 1-40.

35. Gao, C., et al., Troxerutin abrogates ischemic/reperfusion-induced brain injury through ameliorating oxidative stress and neuronal inflammation by inhibiting the expression of NLRP3 in sprague dawley rats. Journal of Environmental Pathology, Toxicology and Oncology, 2021. 40(4).

36. ŞEKERCİ, Ç., et al., Protective Effects of Ellagic Acid on Testicular Ischemia-Reperfusion Injury in Rats. Journal of Urological Surgery, 2023. 10(2).

37. Ketterer, B., Detoxication reactions of glutathione and glutathione transferases. Xenobiotica, 1986. 16(10-11): p. 957-973.

38. YÜKSEL, T.N., et al., Effect of PDE 5 Inhibitor-Avanafil on Renal Ischemia/Reperfusion Injury in Rats. Namık Kemal Tıp Dergisi, 2023. 11(3).

39. Rezq, S., R. Hassan, and M.F. Mahmoud, Rimonabant ameliorates hepatic ischemia/reperfusion injury in rats: Involvement of autophagy via modulating ERK-and PI3K/AKT-mTOR pathways. International immunopharmacology, 2021. 100: p. 108140.

40. Wang, L., et al., The activation of SIRT3 by dexmedetomidine mitigates limb ischemia-reperfusion–induced lung injury. Annals of Translational Medicine, 2022. 10(6).

41. Keskin, M., Kronik karaciğer hastalıkları ile serum paraoksonaz aktivitesi arasındaki ilişki. 2004.

42. Li, H.-L., D.-P. Liu, and C.-C. Liang, Paraoxonase gene polymorphisms, oxidative stress, and diseases. Journal of molecular medicine, 2003. 81: p. 766-779.

43. Tang, W.W., et al., Clinical and genetic association of serum paraoxonase and arylesterase activities with cardiovascular risk. Arteriosclerosis, thrombosis, and vascular biology, 2012. 32(11): p. 2803-2812.

44. Trocha, M., et al., Sitagliptin‐Dependent Differences in the Intensity of Oxidative Stress in Rat Livers Subjected to Ischemia and Reperfusion. Oxidative medicine and cellular longevity, 2019. 2019(1): p. 2738605.
